# Spontaneous coronary artery dissections and associated predisposing factors: a narrative review

**DOI:** 10.1007/s12471-019-1235-4

**Published:** 2019-01-25

**Authors:** E. B. N. J. Janssen, P. W. de Leeuw, A. H. E. M. Maas

**Affiliations:** 1 Radboud University, Nijmegen, The Netherlands; 2 Department of Internal Medicine, Maastricht University Medical Centre (MUMC+), Maastricht, The Netherlands; 3 Department of Cardiology, Radboud University Medical Centre, Nijmegen, The Netherlands

**Keywords:** Spontaneous coronary artery dissection, Acute coronary syndrome, Myocardial infarction, Fibromuscular dysplasia, Women

## Abstract

Spontaneous coronary artery dissection (SCAD) represents around 25% of cases of acute coronary syndromes (ACS) in women aged 40–65 years who have few or no traditional cardiovascular risk factors. It is assumed that the incidence is underestimated, as the angiographic appearance of SCAD may often mimic atherosclerosis. This review aims to examine SCAD by focusing on the associated predisposing factors and precipitating stressors in this heterogeneous patient population, as well as the best treatment approach and the prognosis. Progressive knowledge has improved our current understanding of SCAD, but more awareness among clinicians is necessary. Recently, two position papers from the European Society of Cardiology (ESC) and the American Heart Association (AHA) have been released, which will be summarised in brief.

## Introduction

**Fig. 1:**
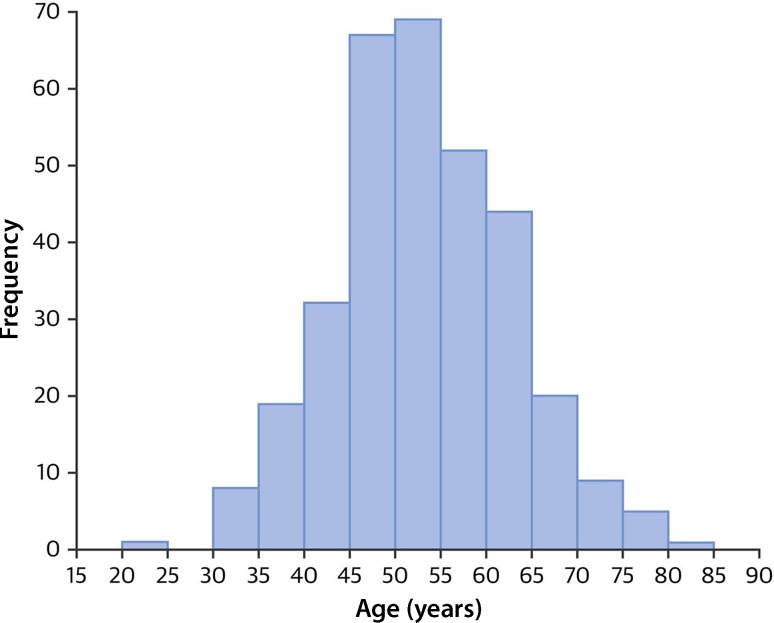
Age distribution of patients affected by spontaneous coronary artery dissection (SCAD); the mean age of patients with SCAD was 52.5 ± 9.6 years, 90.5% of SCAD patients were 65 years of age or younger. (From Saw et al. [1])

Spontaneous coronary artery dissection (SCAD) is an increasingly acknowledged cause of acute coronary syndromes (ACS) in women aged 40–65 years, with a peak around 53 years of age (Fig. 1; [1]). In most cases no or few traditional cardiovascular risk factors are present, although in one third of patients hypertension is reported [1–3]. It is estimated that up to 25% of all ACS in this age group are caused by SCAD, although the diagnosis is often missed [2, 4–7]. Overall, it is estimated that SCAD accounts for 1.7–4% of all ACS and 0.5% of sudden cardiac deaths [4, 8, 9]. SCAD is far more prevalent than initially thought, and pregnancy-associated SCAD accounts for a maximum of 10% of cases. In all patient series there is a very strong female predominance of more than 90%, up to 94% [1, 7, 10–13]. Patients affected by SCAD are heterogeneous with a variety of underlying predisposing factors and precipitating triggers for the event.

## Pathophysiology

**Fig. 2:**
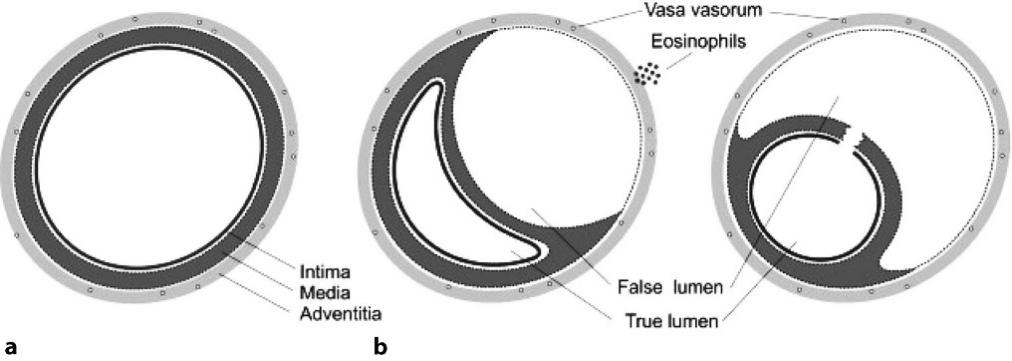
Schematic figure of the two proposed mechanisms of spontaneous coronary artery dissection, **a** Normal artery, **b** intraluminal haemorrhage, **c** intimal tear. (From Saw et al. [15])

SCAD is defined as a spontaneous tear in the coronary arterial wall due to non-atherosclerotic and non-iatrogenic causes [1, 7, 14, 15]. The underlying pathophysiology of SCAD is multifactorial, related to underlying arteriopathies, inflammation, hormonal factors and mixed connective tissue diseases, whereas the acute event is often preceded by emotional or physical triggers [7]. Two mechanisms of SCAD are proposed (Fig. 2): first, a spontaneous separation of the coronary arterial wall caused by an intimal tear and resulting in medial dissection, haemorrhage and subsequently the formation of a false lumen [15, 16]; second and less often, haematoma formation in the media causing separation of two arterial layers and, thereby, leading to the formation of a false lumen and dissection of the true lumen [15, 16]. Coronary blood flow in SCAD is compromised either directly by the intimal tear or indirectly by compression of the medial haematoma on the coronary artery [7, 16]. Until now it is uncertain whether a single dominant mechanism causes SCAD or whether both causal events play a role [12]. In more than 50% of cases the left anterior coronary artery is the affected artery [10, 17].

## Triggers for spontaneous coronary artery dissection

Most cases of SCAD occur in previously healthy young women. Whereas physical stressors are well known, an increasing number of patients report a period of severe emotional distress preceding the acute SCAD event [18]. Research has revealed that severe emotional distress is a potential trigger of an ACS in both women and men [18]. Both emotional and physical stressors create an increase in shear stress which may act as a trigger for dissection [1, 19]. In a study by Saw et al. [1] in 327 SCAD patients, 62% reported potential precipitating stressors prior their SCAD event, with emotional and physical stressors occurring in 48% and 28% of cases, respectively [1].

Emotional precipitating stressors are more common in women than in men [5, 19, 20]. Gender differences in coping with stressful situations may be an important reason why an ACS caused by SCAD is disproportionately predominant in women [21]. Alipour et al. [22] demonstrated that job-related stressors were the most frequently occurring emotional precipitants of SCAD (41%), followed by death of a loved one (21%), arguments (16%), relationship breakdown (15%) and relocation (10%). One working hypothesis in SCAD patients is that (prolonged) emotional distress leads to endothelial dysfunction, low thresholds for vascular spams and elevated levels of catecholamines. This may increase the vulnerability for a sudden intimal tear in the vessel wall.

In male patients provoking triggers are more often related to extreme physical exercise, such as competitive mountain biking or weight lifting [19]. Although less common, retching, vomiting, straining with bowel movement and heavy coughing are also reported [19]. Physical precipitating stressors may cause temporary increases in intra-thoracoabdominal pressure leading to a transient rise in coronary arterial wall pressure [19, 23].

## Predisposing factors

Although the pathophysiology of SCAD is heterogeneous, it is increasingly acknowledged that several predisposing factors increase the susceptibility for SCAD [12]. Predisposing factors which are associated with SCAD are fibromuscular dysplasia (FMD), female gender, pregnancy-related factors, possibly hormonal therapy, mixed connective tissue disorders and inflammatory disorders [19].

The arteriopathy FMD is an associated disease and the most commonly observed associated factor in these patients [1, 19, 23]. FMD is a non-atherosclerotic, non-inflammatory disease of the musculature of small and medium-sized arteries, leading to stenoses, occlusions, aneurysms or dissections of affected arteries [7, 24, 25]. Like SCAD, FMD has a strong female predominance (>80%) [26] and commonly affects the renal and carotid arteries [7, 24]. Two dominant types of FMD are nowadays distinguished which angiographically appear as a tubular (unifocal) lesion and as a string-of-beads (multifocal) lesion respectively [27, 28]. Prior research has found a strong and dominant association of FMD with SCAD with a wide range of 25–86%, which might be due to selection bias or the modality and extent of FMD screening [1, 7, 10, 17, 19, 29–31].

Female gender, pregnancy-related factors and hormonal therapy are also associated with SCAD, suggesting a role for female sex hormones in the pathogenesis of SCAD [12, 17, 32–37]. It is believed that female sex hormones, especially progesterone, which are high in premenopausal women and during pregnancy, influence the integrity of arterial walls [12, 13, 17, 32–37]. However, the precise underlying mechanisms remain unknown.

Although uncommon, mixed connective tissue disorders (MCTD) predispose for SCAD, since these are associated with arterial fragility and vascular dissection [1, 7, 13, 17, 19, 23]. The most common SCAD-associated MCTD are Loeys-Dietz syndrome, Ehlers-Danlos syndrome type 4 and polycystic kidney diseases [1, 7, 23]. Even though only a small proportion of SCAD patients have MCTD (≤5%), its diagnosis remains important as it may guide monitoring, management and familial screening [11].

Systemic inflammatory disorders associated with SCAD are systemic lupus erythematosus, Crohn’s disease, ulcerative colitis, rheumatoid arthritis and coeliac disease [1, 7]. In SCAD infiltrated eosinophils are frequently identified in the adventitia of dissected arteries [38–40]. Whether SCAD is the consequence or the cause of this phenomenon is uncertain.

## Clinical diagnosis of spontaneous coronary artery dissection

A SCAD presents slightly more often as an ST-elevation myocardial infarction (STEMI) than as a non-STEMI. The clinical picture is consistent with the classical type 1 ACS in most cases. Like any other, the initial diagnosis rests on symptoms, ST-T changes and high sensitivity troponin elevations. At coronary angiography, a SCAD may be easily detected. However, typical angiographic SCAD features can be lacking, which is an important reason why SCAD so often goes unrecognised. The false lumen in SCAD is not always clearly visible on angiographic examination and, in addition, SCAD can mimic atherosclerosis [23]. Accurate diagnosis of SCAD is important to provide optimal treatment in the acute phase and thereafter (see next section).

**Fig. 3:**
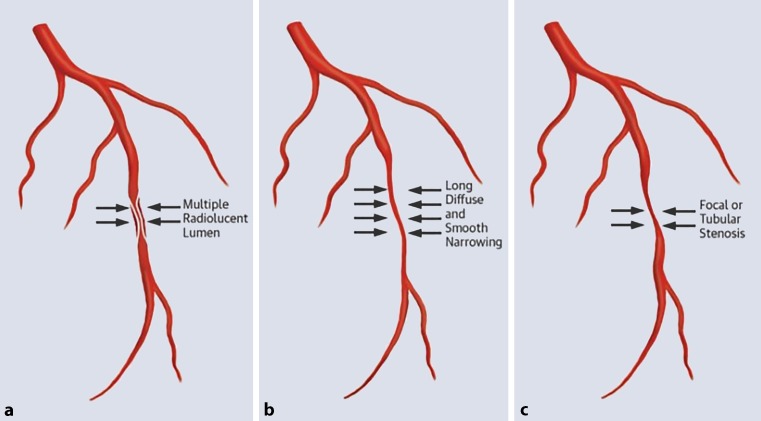
Angiographic spontaneous coronary artery dissection classification system proposed by Saw et al. [1]

To improve diagnosis, Saw et al. [1] have proposed an angiographic classification in which three main angiographic features of SCAD are presented (Fig. 3). In type 1 the classic appearance of contrast dye staining of the arterial wall with multiple radiolucent lumina is seen [1]. Type 2 appears as a long diffuse and smooth narrowing which varies in severity [1]. This overlaps the ‘stick insect’ appearance as mentioned in the supplement of the ESC position paper 2018 [12]. Type 3 angiographic SCAD features focal or tubular stenosis and often mimics atherosclerosis [1]. With intracoronary imaging techniques like optical coherence tomography (OCT) and intravascular ultrasound (IVUS) the diagnosis can often be confirmed, but these interventions pose a serious risk for iatrogenic dissection.

## Acute and chronic treatment

Current guidelines for ACS are inappropriate when an ACS is caused by SCAD. As randomised controlled trials for optimal treatment of SCAD are lacking, current recommendations are based on patient series and expert opinions [12, 13].

**Fig. 4:**
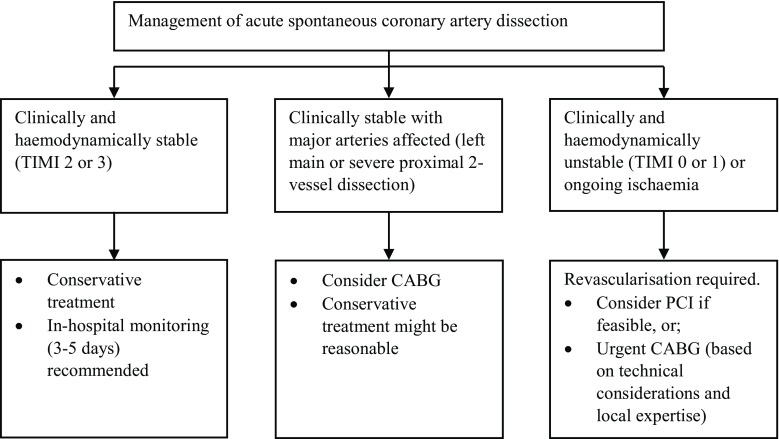
Algorithm for the management of an acute spontaneous coronary artery dissection. *CABG* coronary artery bypass graft, *PCI* percutaneous coronary intervention. (Adapted from a scientific statement of the American Heart Association [13])

In SCAD patients who are clinically and haemodynamically stable (TIMI score 2 or 3) an initial conservative treatment approach is preferred [12, 13, 17, 19, 23, 41–43]. Most (70–97%) SCAD lesions heal spontaneously within several weeks [12, 13]. To avoid iatrogenic damage in the already dissected and vulnerable arteries during percutaneous coronary intervention (PCI), this revascularisation technique is not recommended. In experienced hands intracoronary imaging with OCT/IVUS can be performed to establish the correct diagnosis. Although the majority of conservatively treated SCAD patients have an unremarkable in-hospital course, an important minority of approximately 10% may experience extension of dissection, which may be an indication for acute revascularisation [12, 13, 19, 41]. Therefore, prolonged in-hospital patient monitoring (3–5 days) after the initial event is recommended in conservatively treated patients [12, 13]. In SCAD patients who are clinically or haemodynamically unstable (TIMI score 0 or 1), who show ongoing ischaemia or in whom major arteries are affected, revascularisation either by PCI or coronary artery bypass graft is required [1, 12, 13, 17, 19, 42]. Although evidence-based guidelines are lacking, the AHA recently published a treatment algorithm for SCAD treatment (Fig. 4).

As far as follow-up is concerned, the efficacy of standard ACS medical therapy in SCAD patients is still a matter of debate [14]. Only the use of beta-blockers may lower the risk of recurrence, although the ‘evidence’ is based only on limited retrospective data [1]. In patients with fluctuating residual symptoms the use of diltiazem can be very effective [12]. If patients suffer from hypertension, this should be treated adequately to prevent the recurrence of SCAD. Prolonged use of acetylsalicylic acid after conservative treatment is advised, although the optimal duration of treatment is uncertain. The use of dual antiplatelet therapy should be avoided if no PCI is performed, as this may cause severe menstrual bleeding in premenopausal women. Prolonged use of statins is not advised when lipid levels are normal.

## Secondary prevention

A ‘one size fits all’ approach is not appropriate in secondary prevention of SCAD. Instead, a more patient-tailored prevention program should be offered, including medical management, cardiac rehabilitation, stress management and recommendations regarding physical activity, psychosocial support, contraceptive use and pregnancy if needed [13]. The ESC and AHA recommend SCAD patients to avoid extreme, isometric and competitive physical exercise and to consider psychosocial support [13]. Pregnancy in SCAD survivors requires critical consideration as pregnancy itself is a risk factor and delivery may confer additional physical precipitating distress. Currently, pregnancy is usually not discouraged when the initial event did not cause severe harm and left ventricular function is sufficient. Besides a tailored prevention program, the identification of predisposing factors and precipitating stressors may guide further preventive strategies.

## Prognosis

A recurrent SCAD should be differentiated from extension of a previous dissection and from de novo SCAD. Extension of dissection accounts for 10% of all recurrent SCAD events and usually occurs within 30 days after the initial event [41, 44]. A majority of 90% of recurrent SCAD events is caused by de novo SCAD, usually occurring beyond 30 days after the initial SCAD [41, 44]. Overall, patient series have reported that recurrent de novo SCAD occurs in 12–27% of SCAD patients, depending on the duration of follow-up [1]. To date, risk factors for recurrence remain to be further elucidated. Hypertension and coronary tortuosity are presumably associated with a higher recurrence risk [1, 13]. Whether coronary tortuosity confers a direct risk or appears as a coronary manifestation of FMD is currently unclear [13, 45].

## Future directions

Although our understanding of SCAD has increased enormously over past decades, more knowledge is needed regarding predisposing factors, diagnostics and therapy. Besides, the identification of predisposing stressors will help to improve secondary prevention.

The ESC is currently organising a SCAD registry within the European Observational Research Platform (EOSP) [12]. This registry is expected to start in spring 2019. Within the EORP-SCAD registry genetic studies will be conducted as well.

Presumably, the currently most important task in improving cardiovascular care for SCAD patients is to increase awareness and suspicion among clinicians in the field of cardiology, especially for ACS in younger women. This may be a first step forward in improving survival in this growing patient population.
